# Alpha1-Antitrypsin Attenuates Renal Fibrosis by Inhibiting TGF-β1-Induced Epithelial Mesenchymal Transition

**DOI:** 10.1371/journal.pone.0162186

**Published:** 2016-09-08

**Authors:** Jang-Hee Cho, Hye-Myung Ryu, Eun-Joo Oh, Ju-Min Yook, Ji-Sun Ahn, Hee-Yeon Jung, Ji-Young Choi, Sun-Hee Park, Yong-Lim Kim, Ihm Soo Kwak, Chan-Duck Kim

**Affiliations:** 1 Department of Internal Medicine, Kyungpook National University School of Medicine, Daegu, Korea; 2 Department of Internal Medicine, Pusan National University Hospital, Busan, Korea; University of Louisville, UNITED STATES

## Abstract

Alpha1-antitrypsin (AAT) exerts its anti-inflammatory effect through regulating the activity of serine proteinases. This study evaluated the inhibitory effects of AAT against the transforming growth factor (TGF)-β1 induced epithelial-to-mesenchymal transition (EMT) in unilateral ureter obstruction (UUO) mice and Madin-Darby canine kidney (MDCK) cells. C57BL/6 mice with induced UUO were injected intraperitoneally with AAT (80 mg/Kg) or vehicle for 7 days. MDCK cells were treated with TGF-β1 (2 ng/mL) for 48 hours to induce EMT, and co-treated with AAT (10 mg/mL) to inhibit the EMT. Masson’s trichrome and Sirius red staining was used to estimate the extent of renal fibrosis in UUO mice. The expression of alpha-smooth muscle actin (α-SMA), vimentin, fibronectin, collagen I, and E-cadherin in MDCK cells and kidney tissue were evaluated. Masson’s and Sirius red staining revealed that the area of renal fibrosis was significantly smaller in AAT treated UUO group compared with that of UUO and vehicle treated UUO groups. AAT treatment attenuated upregulation of Smad2/3 phosphorylation in UUO mouse model. Co-treatment of MDCK cells with TGF-β1 and AAT significantly attenuated the changes in the expression of α-SMA, vimentin, fibronectin, collagen I, and E-cadherin. AAT also decreased the phosphorylated Smad3 expression and the phosphorylated Smad3/Smad3 ratio in MDCK cells. AAT treatment inhibited EMT induced by TGF-β1 in MDCK cells and attenuated renal fibrosis in the UUO mouse model. The results of this work suggest that AAT could inhibit the process of EMT through the suppression of TGF-β/Smad3 signaling.

## Introduction

Progressive renal fibrosis is considered as the final pathway of chronic kidney disease regardless of its etiology [1]. Among the effector cells contributing to the development of renal fibrosis, myofibroblasts originating from the tubular epithelial cells play an essential role [2]. They accumulate in the tubular interstitial area and progress through the epithelial-to-mesenchymal transition (EMT). During the EMT process, tubular epithelial cells lose their adhesion molecules such as E-cadherin, and gain the mesenchymal cell marker, alpha-smooth muscle actin (α-SMA), vimentin, fibronectin, and collagen I [3]. EMT further induces tubular destruction and atrophy resulting in the deterioration of renal function [4,5].

Transforming growth factor (TGF)-β1, one of the most important regulating factors for renal fibrosis, promotes trans-differentiation of tubular epithelial cells into α-SMA-expressing myofibroblasts [6]. TGF-β1 modulates the progression of renal fibrosis and induces renal scarring by activation of the Smad signaling pathway [7]. Several molecules blocking the phenotypic transition have been reported to reverse the EMT and attenuate renal fibrosis [8,9,10]. These studies suggest that interventions to reverse or inhibit the EMT might be a potential treatment strategy to manage chronic kidney disease.

Alpha1-antitrypsin (AAT) is a member of the serpin superfamily and major serine proteinase inhibitor in circulation [11]. AAT is a potent inhibitor of multiple serine proteinases with high activity against the neutrophil serine proteinases, neutrophil elastase, and proteinase-3 [12,13]. AAT plays an important role in protecting tissue injury triggered by proteinases, especially in conditions of inherited AAT deficiency such as chronic obstructive pulmonary disease. However, mounting evidence indicates that AAT has anti-inflammatory and tissue-protective properties, independent of proteinase inhibition. In experimental models, AAT suppressed the release of neutrophil chemokines [14], prevented tumor necrosis factor (TNF)-induced apoptosis [15], and reduced superoxide production by neutrophils [16].

While these effects demonstrate the anti-inflammatory and tissue-protective properties of AAT, the exact underlying mechanism has not been fully investigated. We hypothesized that AAT exerts its anti-inflammatory effect through modulating TGF-β1 pathway, which in turn, might inhibit renal fibrosis. The present study investigated the protective effect of AAT on TGF-β1 mediated EMT in the unilateral ureteral obstruction (UUO) renal fibrosis animal models and in Madin-Darby canine kidney (MDCK) cells.

## Materials and Methods

### Animal experiments

#### UUO animal model

Male C57BL/6 mice (Samtako, Osan, Korea), 8 weeks of age, had free access to standard food and water throughout the experiments. Mice were anesthetized with an intraperitoneal injection of pentobarbital sodium (50 mg/Kg). After a left flank incision, left ureter was isolated and ligated with 6–0 silk to make UUO model. Control group of mice underwent sham operation, which was identical to that of UUO group, except the ligation of the ureter. The experimental protocols for animal models were reviewed and approved by the boards of the Kyungpook National University and Kyungpook National University Hospital (KNU-2015-0041).

#### Experimental protocol for UUO animal model

The mice were divided into 4 groups. Groups 1 and 2 were sham-operated mice (n = 4) and UUO mice (n = 8), respectively. Additional 18 UUO mice were randomly assigned to either the vehicle treated group (group 3; n = 8) or AAT (Aralast^®^; Baxter Healthcare, Vienna, Austria) treated group (group 4; n = 10). In group 3 mice, 0.9% normal saline was administered every day from the day of operation. Group 4 mice were administered AAT intraperitoneally at the dose of 80 mg/Kg body weight every day from the day of operation to day 7. All mice were sacrificed 7 days after the initial surgery, under anesthesia of isoflurane (Hana Pharma Corp., Kyonggi-Do, Korea).

#### Assessment of renal fibrosis area

Kidney sections of 2 μm thickness were stained with Masson’s trichrome and Sirius Red for examination of under a light microscope at 200× magnification. Collagen deposition was quantified using the i-solution DT image software (IMT i-solution, Vancouver, BC, Canada) in more than 5 randomly selected fields in the sections of cortex and medulla.

#### Immunohistochemistry

Kidney tissues from each experimental group were immersion-fixed with 4% paraformaldehyde (pH 7.4) and the sections were embedded in paraffin. Immunohistochemical staining was performed using by anti-α-SMA (1:100; Abcam, Cambridge, UK), anti collagen I (1:200; Abcam), anti Fibronectin (1:100; Abcam) and anti-E-cadherin (1:200; BD Biosciences, Bedford, MA, USA) antibodies and then detected by the EnVision-HRP kit (Dako, Carpinteria, CA, USA). All sections were counterstained with Mayer’s hematoxylin. The immunolabeling was examined under a Leica DM IRB inverted microscope (Leica Microsystems, Wetzlar, Germany) equipped with a CoolSNAP HQ camera (Photometrics, Tucson, AZ, USA).

#### Immunoblotting of EMT markers and Smad2/3 in kidney

Immunoblotting using kidney tissues was done with primary antibodies against α-SMA (1:10000; Sigma), fibronectin (1:5000; Abcam), collagen I (1:1000; Abcam), E-cadherin (1:5000; BD Biosciences), Smad2/3 (1:2000; BD Biosciences), and phosphorylated Smad2/3 (p-Smad2/3; 1:1000; Abcam). The sites of the antigen-antibody reaction were detected with horseradish peroxidase conjugated secondary antibodies (P447, diluted 1:10000; Dako, Glostup, Denmark) using an enhanced chemiluminescence (ECL) advanced detection system (GE Healthcare, Little Chalfont, UK). The band densities were quantified by densitometry and compared to the expression of glyceraldehyde-3-phosphate dehydrogenase (GAPDH). Computer analysis of band pixel intensities was performed by Scion Image (Scion, Frederick, MD, USA).

#### RNA isolation and complementary DNA synthesis

Total RNA was extracted from kidney tissues with TRI Reagent (Molecular Research Center Inc., Cincinnati, OH, USA). RNA concentration and purity were determined using Nano Drop ND-1000 (Nano-Drop Technologies, Wilmington, DE, USA). One microgram of total RNA was transcribed reversely by using the PrimeScript cDNA Synthesis kit (Takara Shuzo Co., Otsu, Japan).

#### Real Time Reverse Transcriptase-Polymerase Chain Reaction (RT-PCR)

All primers for real time RT-PCR were designed by using Primer (Table 1) Express V1.5 software (Applied Biosystems, Foster City, CA, USA). Real time RT-PCR for α-SMA, E-cadherin, collagen I and β-actin was done in duplicate for each sample. The PCR reaction comprised pre-denaturation at 50°C for 2 min and 95°C for 10 min, followed by 40 cycles at 95°C for 15 s and 60°C for 1 min. The results were normalized to the corresponding ΔCt values for the control primer set of β-actin mRNA, for which, fold enrichment was calculated as 2 –[ΔCt (assayed gene)–ΔCt (β-actin)]. The relative changes in assayed gene with respect to control, mRNA/β-actin mRNA ratio, for both control and experimental groups were determined by the formula 2 –ΔΔCt.

**Table 1:** Sequences of real time RT-PCR primers.

Type	Primers	Sequences (5′ to 3′)
Mice	α-SMA (Forward)	CTG ACA GAG GCA CCA CTG AA
	α-SMA (Reverse)	CAT CTC CAG AGT CCA GCA CA
	E-cadherin (Forward)	GCA GTT CTG CCA GAG AAA CC
	E-cadherin (Reverse)	TGG ATC CAA GAT GGT GAT GA
	Collagen I (Forward)	ACA ACC GCT TTG CCA CTT CT
	Collagen I (Reverse)	CGT AAG TCA CGG GCA CGT T
	β-actin (Forward)	GAT CTG GCA CCA CAC CTT CT
	β-actin (Reverse)	CTT TTC ACG GTT GGC CTT AG
MDCK cells	α-SMA (Forward)	TGT TCC AGC CGT CCT TCA T
	α-SMA (Reverse)	GGC GTA GTC TTT CCT GAT G
	E-cadherin (Forward)	TTG AGA CCA CGC AGC AAT ACA C
	Vimentin (Forward)	GCC ATC AAC ACC GAG TTC AA
	Vimentin (Reverse)	GGA AGC GCA CCT TGT CGA T
	Fibronectin (Forward)	GAA CCA CGC CGA ACT ACG AT
	Fibronectin (Reverse)	ATG CGA TAC ATG ACC CCT TCA
	E-cadherin (Reverse)	CAA CCA CGT CCA CAG TGA CTG T
	GAPDH (Forward)	GAT GCC CCC ATG TTT GTG A
	GAPDH (Reverse)	TTT GGC TAG AGG AGC CAA GCA

### In vitro experiments

#### Determination of cell viability

MDCK cells (1.0×10^4^) cultured in a 96-well plate in DMEM medium were treated with AAT (1, 5, 10, 50 mg/mL) for 24, 48, or 72 hours. After the medium was changed at the end of the stipulated time of AAT treatment, MDCK cells were cultured for a further duration of 24 hours and then treated with 500 μg/mL MTT [(3-(4, 5-dimethylthiazol-2yl)-2,5-diphenyltetrazolium bromide; Sigma)] for 4 hours. After melting with 200 μL dimethyl sulfoxide (DMSO) solution (Amresco, Solon, OH, USA), absorbance was estimated at 570 nm with a microplate reader (Bio-Rad Model 550; Hercules, CA, USA).

#### MDCK cell culture

MDCK cells were cultured on six well culture plates for 48 hours in complete medium that contained 10% FBS, using previously described methods [17]. The cells were kept in serum free medium for 24 hours and then placed into medium supplemented with 1% FBS. The cells were treated with (1) only FBS medium, (2) AAT (10 mg/mL), (3) TGF-β1 (Santa Cruz Biotechnology, Santa Cruz, CA, USA; 2 ng/mL), or (4) TGF-β1 (2 ng/mL) + AAT (10 mg/mL) for an additional 48 hours.

#### Immunoblotting of EMT markers and Smad3 and Smad2 in MDCK cells

Immunoblotting using whole-cell lysate was done with primary antibodies against α-SMA (1:5000; Sigma), E-cadherin (1:5000; BD Biosciences), vimentin (1:1000; Abcam), fibronectin (1:2500; Abcam), collagen I (1:2000; Abcam), Smad3 (1:2500; Cell signaling technology, Danvers, MA, USA), and phosphorylated Smad3 (p-Smad3; 1:2500; Cell signaling technology), Smad2 (1:2500; BD Biosciences), and phosphorylated Smad2 (p-Smad2; 1:2500; Millipore). The sites of the antigen-antibody reaction were detected with horseradish peroxidase conjugated secondary antibodies (P447, diluted 1:10000; Dako, Glostup, Denmark) using an enhanced chemiluminescence (ECL) advanced detection system (GE Healthcare, Little Chalfont, UK). The band densities were quantified by densitometry and compared to the expression of glyceraldehyde-3-phosphate dehydrogenase (GAPDH). Computer analysis of band pixel intensities was performed by Scion Image (Scion, Frederick, MD, USA).

#### Quantitative real time RT-PCR of MDCK cells

Total RNA was extracted from MDCK cell lysates with TRI reagent (Molecular Research Center Inc., Cincinnati, OH, USA) according to the manufacturer’s instructions. One microgram of total RNA was reverse transcribed with the PrimeScript cDNA Synthesis kit (Takara Shuzo Co., Otsu, Shiga, Japan). Quantitative real time RT-PCR was performed on the PRISM 7700 Sequence Detection System (Applied Biosystems) using the SYBR green PCR Master Mix (Applied Biosystems) and gene specific primers. The primer sets used in the RT-PCR are summarized in Table 1. Results were calculated by the comparative Ct method for relative quantification of gene expression and normalization with respect to GAPDH expression.

#### Immunocytochemistry

MDCK cells were cultured on chamber slides and fixed with 3.7% paraformaldehyde in PBS for 30 min. The cells were washed with PBS and then permeated with 0.3% Triton for 15 min. The cells were incubated with primary antibodies against α-SMA (1:200) and E-cadherin (1:200) overnight at 4°C. The cells were washed and incubated with the secondary antibodies (Alexa Fluor 488 and Alexa Fluor 594, Life technologies, Eugene, OR, USA) for 2 hours at room temperature. The nucleus was counterstained with 4′, 6-diamidino-2-phenylindole (DAPI) and the slides were mounted with antifade mounting reagent (Molecular Probes). The sections were examined by a Zeiss confocal scanning laser microscope using LSM 5 Exciter (Carl Zeiss, Oberkochen, Germany).

### Statistical Analyses

Data are expressed as mean ± standard deviation. Comparison among groups was made with Student’s *t*-test or one-way ANOVA. Multiple-comparison tests were applied only when the difference as determined by ANOVA was significant (P < 0.05). The statistical analysis was performed using the SPSS system for Windows, version 21.0 (SPSS Inc., Chicago, IL, USA). P values < 0.05 were considered statistically significant.

## Results

### AAT Treatment Attenuated Renal Fibrosis in UUO Kidneys

**Fig 1:**
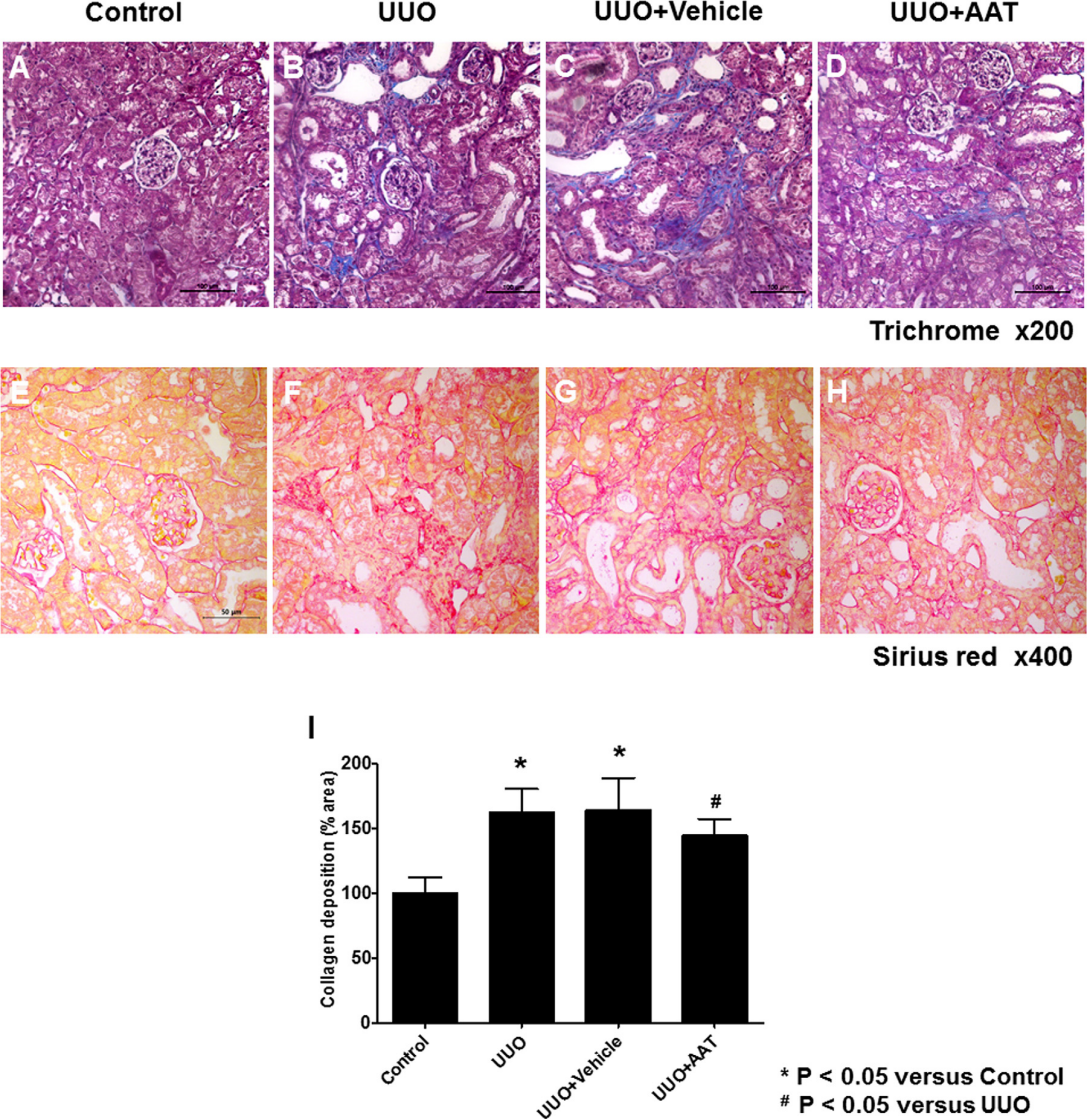
Masson’s trichrome and Sirius red staining for assessing renal fibrosis area at day 7 after unilateral ureteral obstruction (UUO). (A and E) Sham-operation (Control). (B and F) UUO. (C and G) UUO treated with 9% NaCl (UUO + Vehicle). (D and H) UUO treated with 80 mg/Kg AAT (UUO + AAT). (I) Semi-quantitative assessment of renal fibrosis area.

UUO mouse model was used to evaluate the effect of AAT on TGF-β1 induced EMT. We first examined the UUO induced changes in the expressions of collagen by staining kidney sections with Masson’s trichrome and Sirius red. UUO increased the expression of collagen by day 7 compared to that in control kidneys (Fig 1A and 1B). This change was also observed in the vehicle treated UUO kidneys (Fig 1C). In contrast, AAT treatment significantly reduced the collagen expression in UUO kidneys (Fig 1D). Sirius red staining showed AAT treatment significantly attenuated the renal fibrosis in UUO kidneys (Fig 1E to 1H). Semi-quantitative assessment of the renal fibrosis area also revealed that AAT treatment attenuated the progression of fibrosis in UUO kidneys (Fig 1I).

### AAT Treatment Reversed the Increased Expression of Mesenchymal Markers and the Decreased Expression of Epithelial Marker in UUO Kidneys

**Fig 2:**
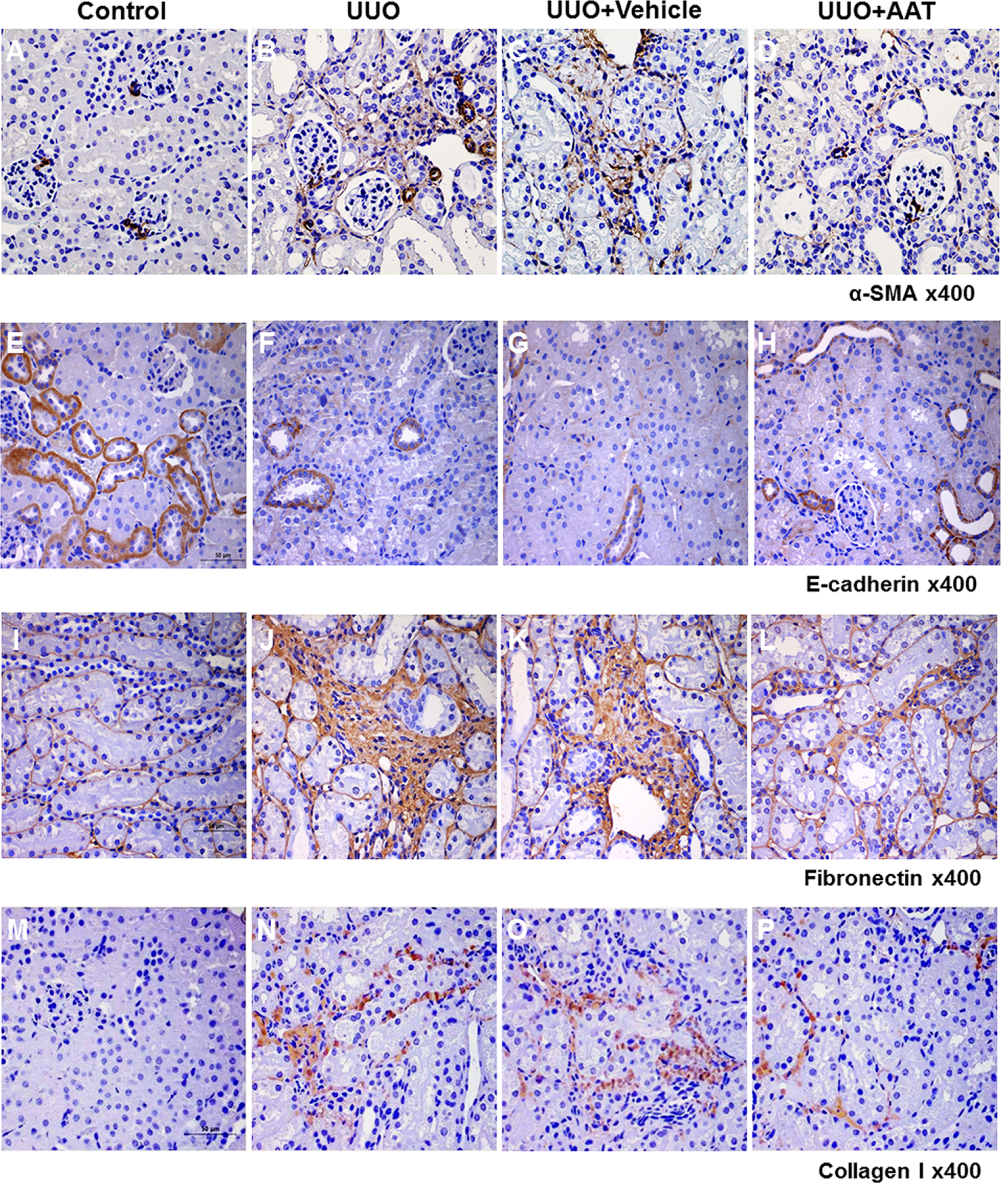
Expression of α-SMA, E-cadherin, fibronectin and collagen I in the kidneys of mice with UUO. Immunoperoxidase microscopy showed increased labeling intensity of α-SMA, fibronectin, and collagen I at the interstitial area in the UUO (B, J, and N) and vehicle treated UUO (C, K, and O) kidneys compared to that in the sham operated control kidneys (A, I, and M). In contrast, UUO (F) and vehicle treated UUO (G) kidneys showed decreased labeling intensity of E-cadherin compared to that in the control kidneys (E). AAT treatment reversed the changes in the labeling intensities of α-SMA, E-cadherin, fibronectin, and collagen I in the UUO kidneys (D, H, L, and P).

We examined the changes in the expressions of α-SMA, collagen I, fibronectin, and E-cadherin in the UUO kidneys. Immunoperoxidase localization revealed increased labeling intensity of α-SMA, collagen I, and fibronectin in the interstitial area in the UUO and vehicle treated UUO kidneys compared to that in the sham operated control kidneys. In contrast, UUO and vehicle treated UUO kidneys showed decreased labeling intensity of E-cadherin compared to that in control kidneys. Intraperitoneal AAT treatment reversed the changes in the labeling intensity of α-SMA, collagen I, fibronectin, and E-cadherin in the UUO kidneys (Fig 2).

**Fig 3:**
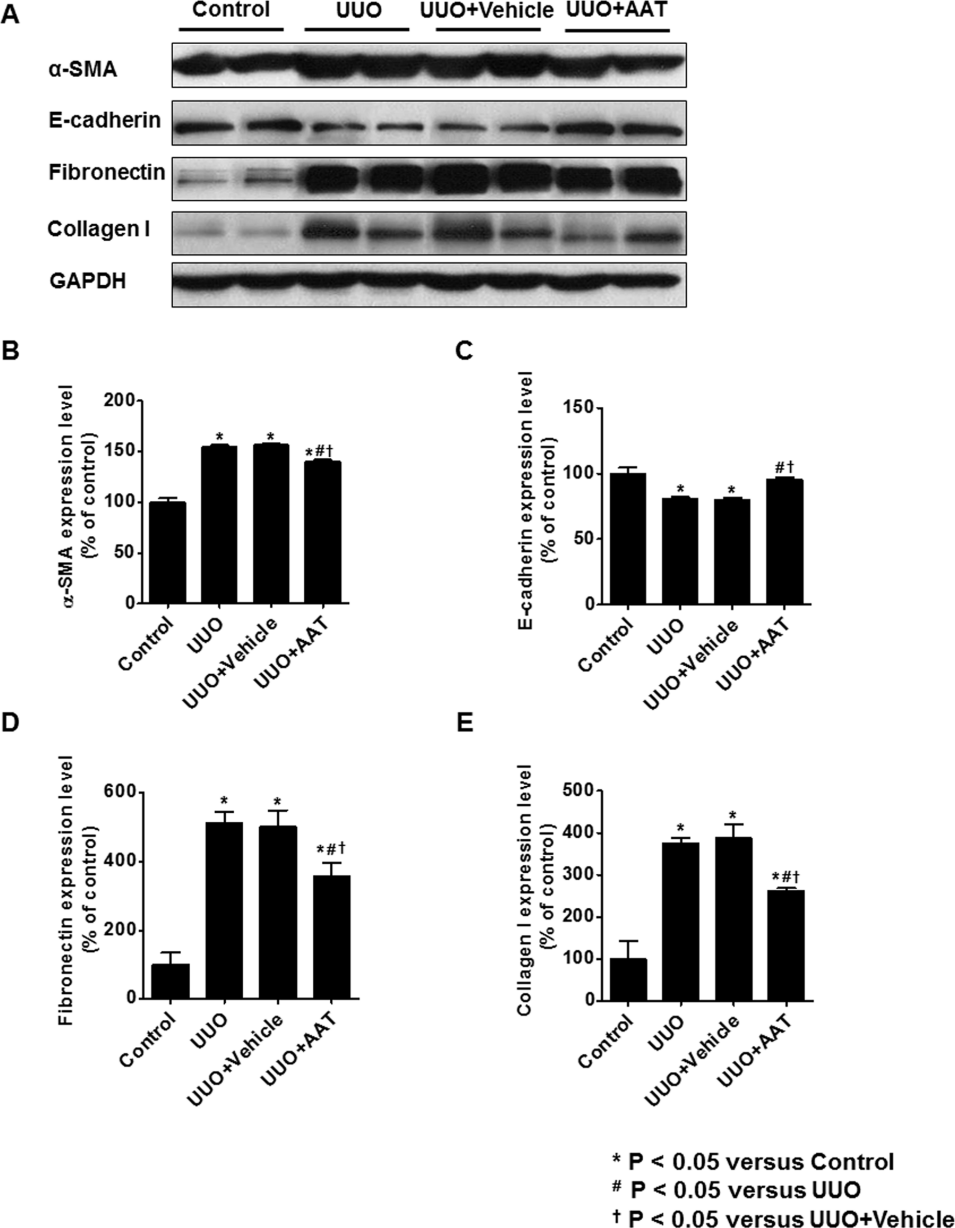
Expression of α-SMA, E-cadherin, fibronectin, and collagen I in UUO kidneys. Expression of α-SMA, fibronectin, and collagen I were upregulated in UUO and vehicle treated UUO kidneys. Treatment with AAT significantly decreased the change in the expression of α-SMA, fibronectin, and collagen I of UUO kidneys. In contrast, the protein level of E-cadherin decreased in UUO and vehicle treated UUO kidneys and increased significantly by AAT treatment.

Expression of α-SMA, fibronectin, and collagen I were upregulated in UUO and vehicle treated UUO kidneys. Treatment with AAT significantly decreased the change in the expression of α-SMA, fibronectin, and collagen I of UUO kidneys. In contrast, the protein level of E-cadherin decreased in UUO and vehicle treated UUO kidneys and increased significantly by AAT treatment (Fig 3).

**Fig 4:**
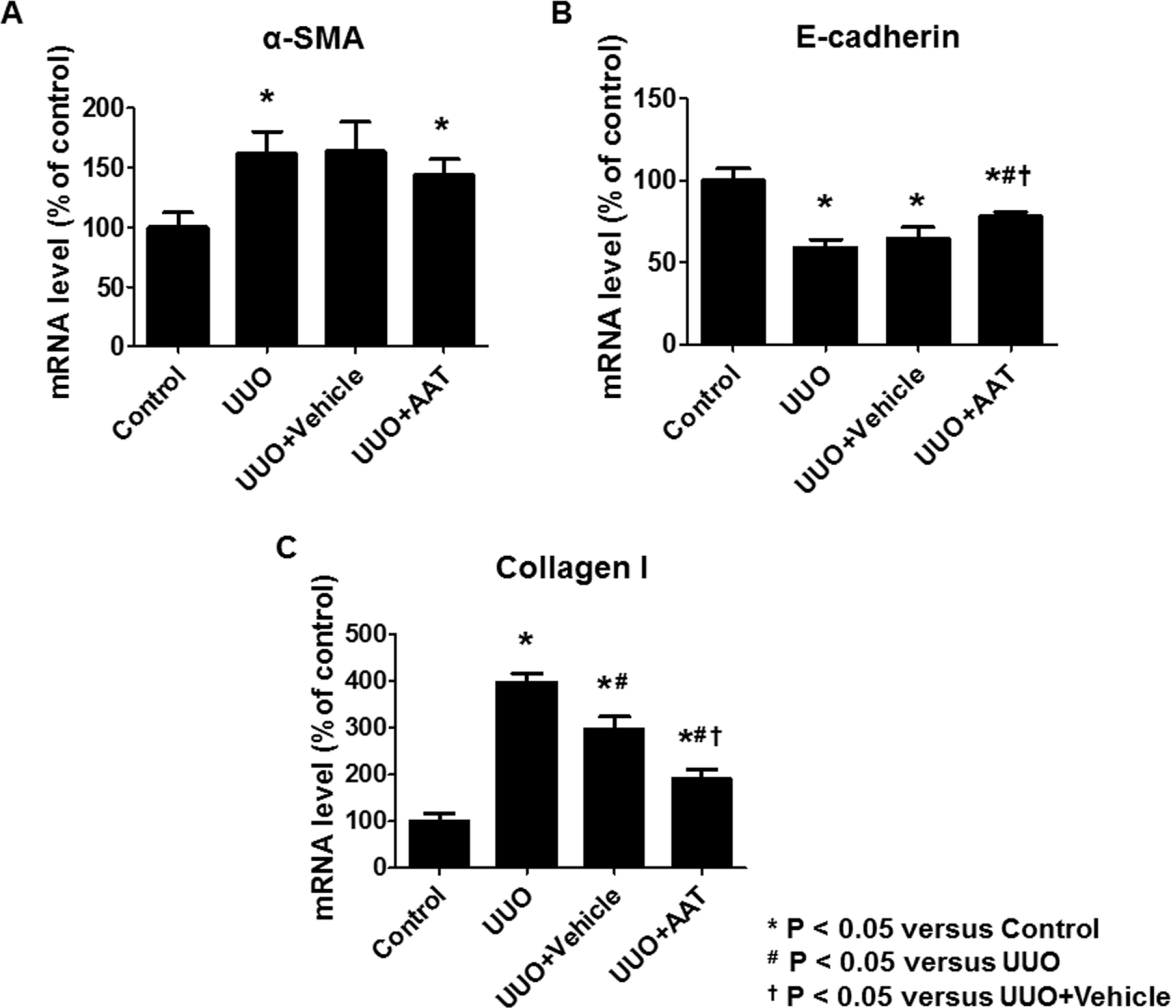
The mRNA level of α-SMA, E-cadherin, and collagen I in kidney tissue. Real time RT-PCR analysis revealed significant increase in the expression of α-SMA and collagen I mRNA and decrease in E-cadherin mRNA expression in the UUO group compared to that in the control group. AAT treatment (UUO + AAT) significantly inhibited the upregulation of collagen I and downregulation of E-cadherin mRNA level compared to that in UUO alone or UUO + Vehicle treatments. AAT treatment also showed a tendency to inhibit the upregulation of α-SMA mRNA levels in the UUO kidneys.

Real time RT-PCR analysis revealed increased expressions of α-SMA and collagen I mRNA and decreased expression of E-cadherin mRNA in the UUO group compared to that in the control group. AAT treatment significantly inhibited the upregulation of collagen I mRNA and downregulation of E-cadherin mRNA expression in the UUO group compared to that in the untreated or the vehicle treated UUO groups. AAT treatment also showed a tendency to inhibit the upregulation of α-SMA mRNA levels in the UUO kidneys (Fig 4).

### AAT Treatment Attenuated Upregulation of Smad2/3 Phosphorylation in UUO Kidneys

**Fig 5:**
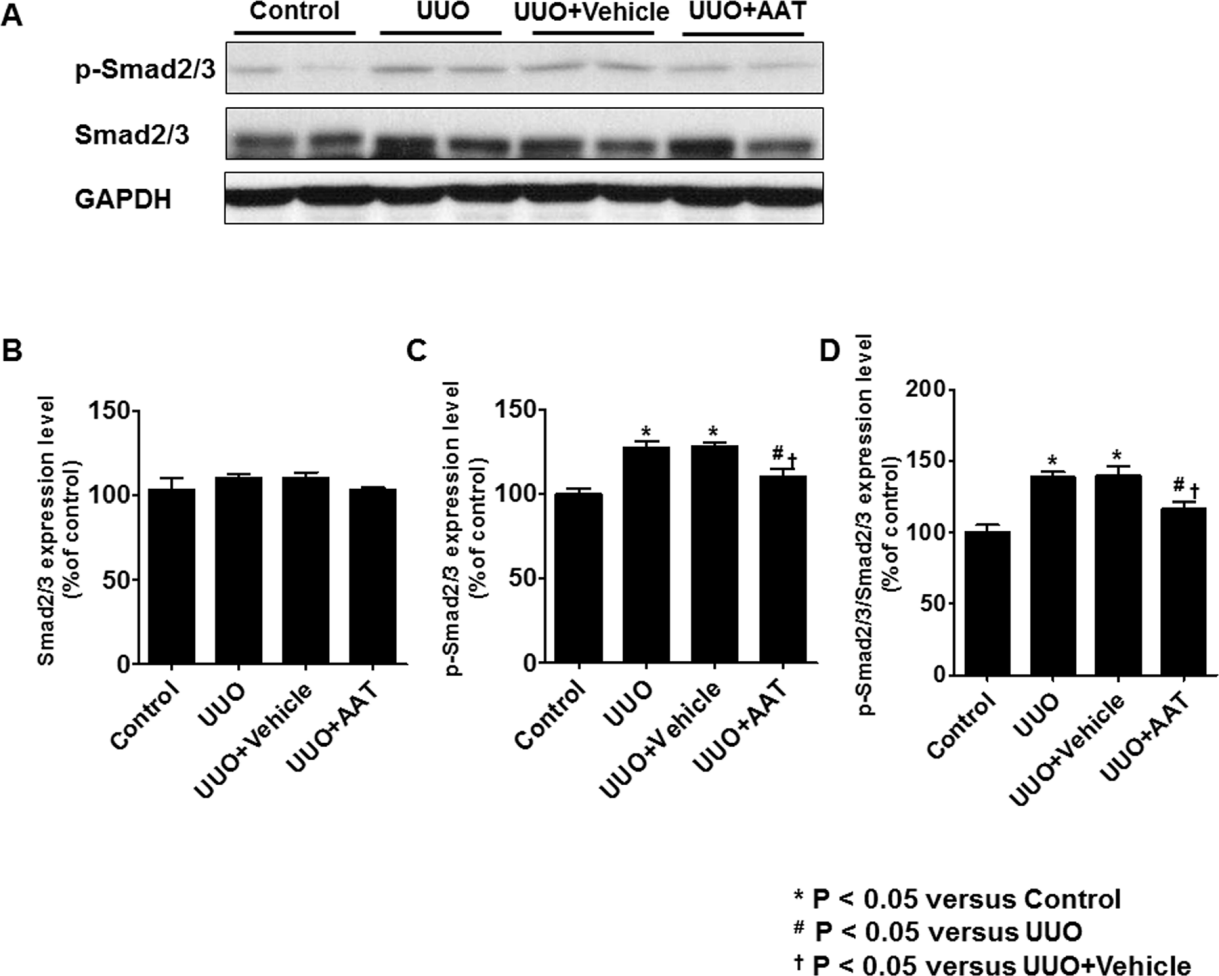
Expression of Smad2/3 and phosphorylated Smad2/3 (p-Smad2/3) in UUO kidneys. The level of p-Smad2/3 and the ratio of p-Smad2/3 to Smad2/3 were significantly increased in the UUO and vehicle treated UUO groups. After treatment with AAT, UUO kidneys showed significant decrease in the level of p-Smad2/3 and the ratio of p-Smad2/3 to Smad2/3 compared to UUO kidneys.

The levels of Smad2/3 and p-Smad2/3 in UUO kidneys were analyzed by immunoblotting. The level of p-Smad2/3 and the ratio of p-Smad2/3 to Smad2/3 were significantly increased in the UUO and vehicle treated UUO groups. After treatment with AAT, UUO kidneys showed significant decrease in the level of p-Smad2/3 and the ratio of p-Smad2/3 to Smad2/3 compared to UUO kidneys (Fig 5).

### Effect of AAT on the MDCK cell Viability

The effect of AAT on the viability of MDCK cells was evaluated by MTT assay. MDCK cells were treated with different concentrations of AAT (1, 5, 10, 50 mg/mL) for 24, 48, or 72 hours. The viability of MDCK cells decreased significantly at the concentration of 50 mg/mL (data not shown). Therefore, subsequent experiments were performed with AAT concentration of 10 mg/mL.

### AAT Treatment Inhibited TGF-β1 Mediated EMT in MDCK cells

**Fig 6:**
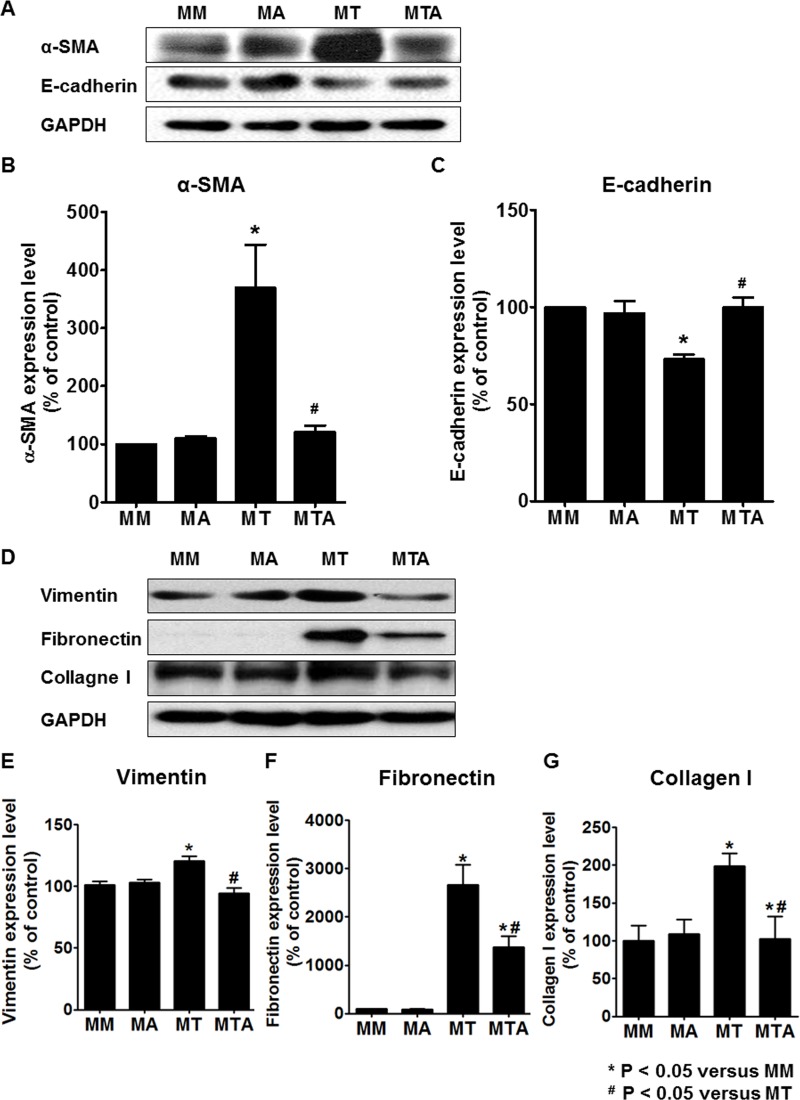
Expression of EMT markers in MDCK cells treated with TGF-β1 and AAT. TGF-β1 exposure increased the expression of α-SMA (A and B) and decreased the expression of E-cadherin (A and C), which were reversed by AAT co-treatment. Vimentin, fibronectin, and collagen I significantly increased after TGF-β1 treatment and decreased after co-treatment with TGF-β1 and AAT (D to G). The data were normalized relative to GAPDH control. MM, cells were cultured in medium for 24 hours and then in fresh medium for additional 48 hours; MA, cells were cultured in medium for 24 hours and then in medium containing AAT for additional 48 hours; MT, cells were cultured in medium for 24 hours and then in medium containing TGF-β1 for additional 48 hours; MTA, cells were cultured in medium for 24 hours and then in medium containing TGF-β1 and AAT for additional 48 hours.

To evaluate whether AAT could protect against TGF-β1 mediated EMT, we examined the effect of AAT on the regulation of EMT markers in MDCK cells. Immunoblots of MDCK cells showed that AAT was associated with significant changes in the level of α-SMA and E-cadherin. We then compared MDCK cells incubated with TGF-β1 and MDCK cells co-treated with TGF-β1 and AAT. TGF-β1 treatment for 48 hours upregulated α-SMA expression at protein level. However, AAT treatment significantly attenuated the TGF-β1 induced upregulation of α-SMA. Vimentin, fibronectin, and collagen I also significantly increased after TGF-β1 treatment and decreased after co-treatment with TGF-β1 and AAT. Expression of E-cadherin protein level in MDCK cells decreased after TGF-β1 exposure, and the reduction was reversed by the simultaneous treatment with AAT (Fig 6).

**Fig 7:**
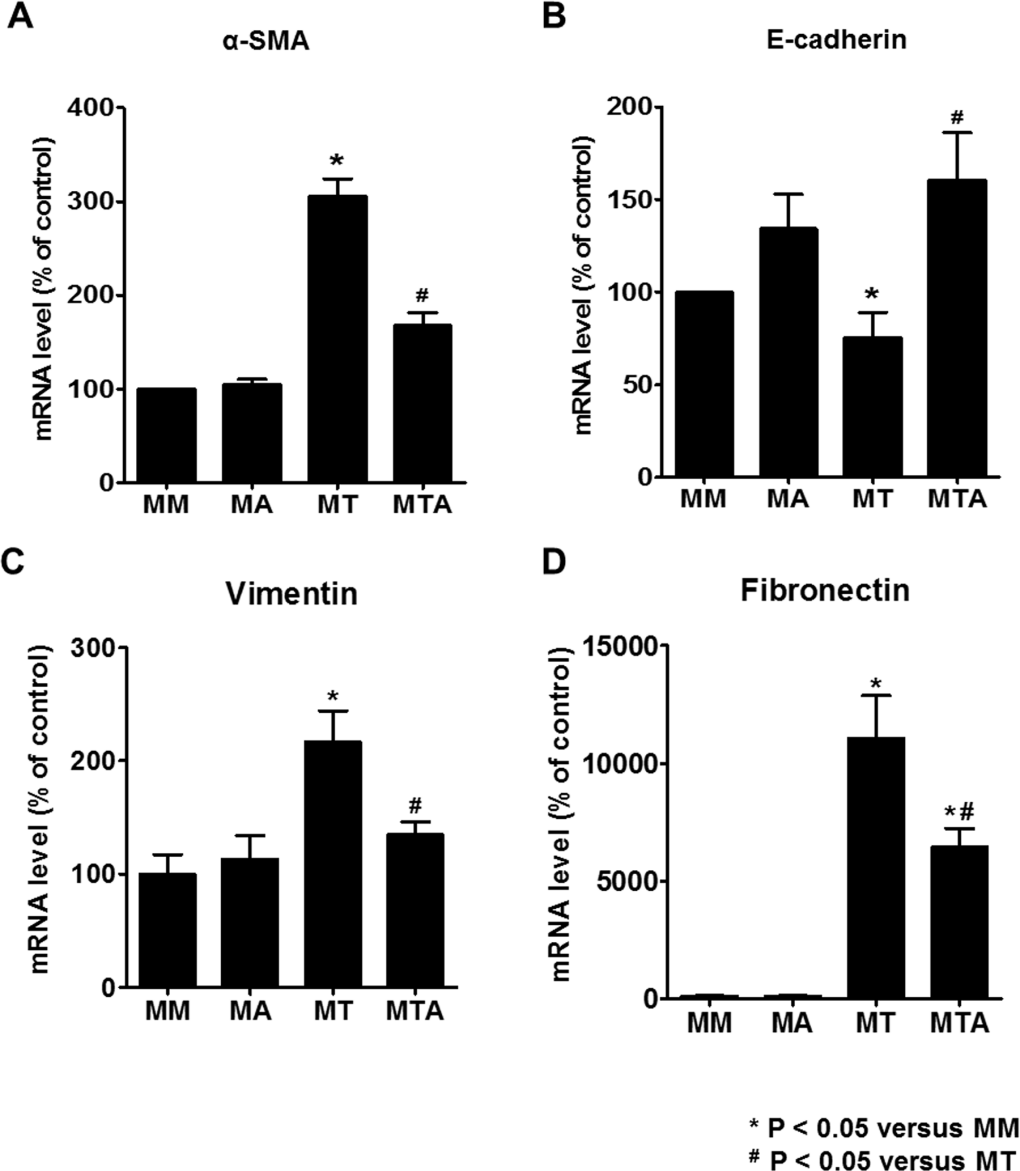
The mRNA level of α-SMA, E-cadherin, vimentin, and fibronectin in MDCK cells. MDCK cells exposed to TGF-β1 showed upregulation of α-SMA, vimentin, and fibronectin mRNA and downregulation of E-cadherin mRNA. Co-treatment with AAT and TGF-β1 ameliorated the changes in the expression of α-SMA, vimentin, fibronectin, and E-cadherin mRNA levels in MDCK cells.

The mRNA level of α-SMA, vimentin, and fibronectin also were upregulated in MDCK cells exposed to TGF-β1. Co-treatment with AAT neutralized the changes in the expression of α-SMA, vimentin, and fibronectin mRNA level. In contrast, the mRNA level of E-cadherin decreased in MDCK cells treated with TGF-β1 and increased significantly by TGF-β1 and AAT co-treatment (Fig 7).

**Fig 8:**
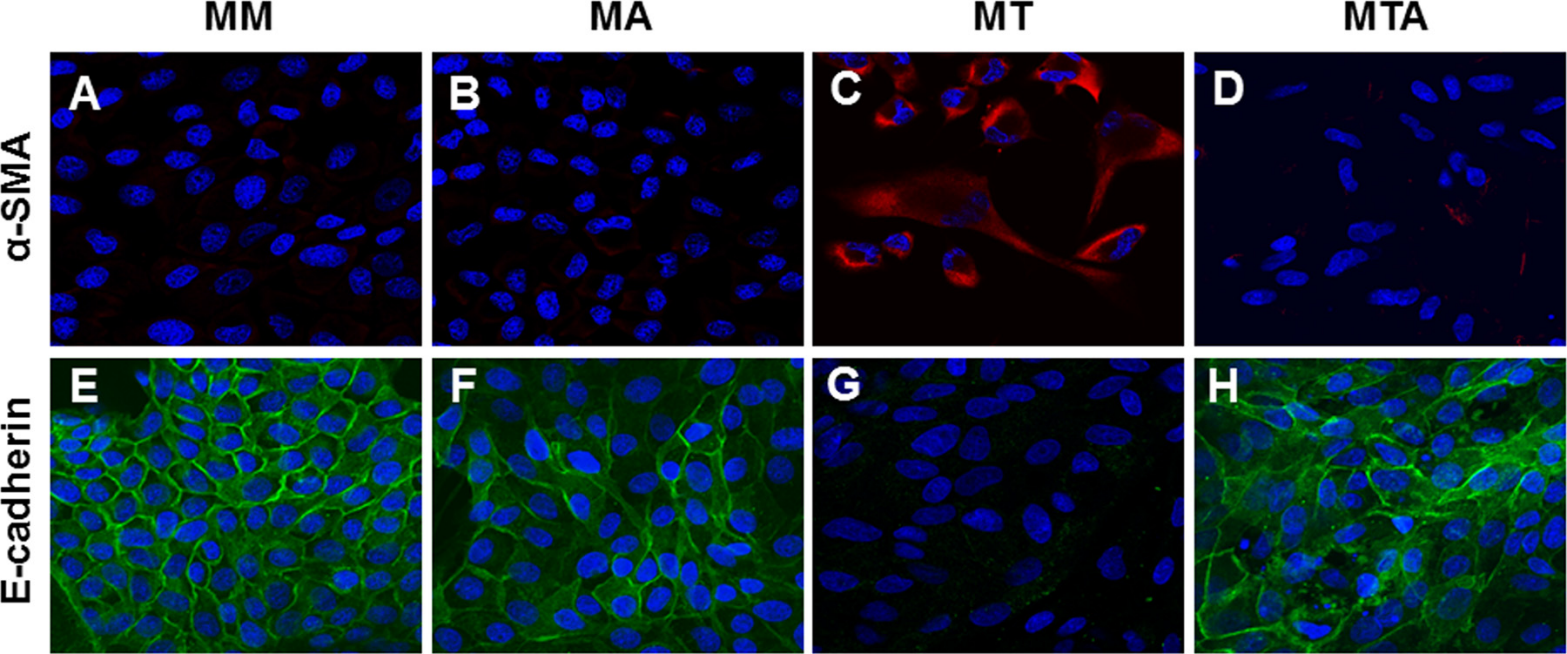
**Immunofluorescence microscopy of α-SMA (A to D) and E-cadherin (E to H) in MDCK cells after TGF-β1 and AAT treatment.** AAT did not cause significant change in MDCK cells cultured in medium alone (B and F). The increased immunolabeling intensity of α-SMA (C, red) seen in response to TGF-β1 treatment was attenuated by AAT treatment (D). The decreased immunolabeling intensity of E-cadherin (G, green) in response to TGF-β1 treatment was augmented by AAT treatment (H). The blue staining (A to H) is nuclear counterstaining with DAPI.

Immunofluorescence findings were consistent with those of immunoblots and real time RT-PCR. AAT treatment did not have any effect on MDCK cells grown in medium devoid of TGF-β1 (Fig 8B and 8F). TGF-β1 exposure increased the labeling intensity of α-SMA (Fig 8C) and decreased the labeling intensity of E-cadherin in MDCK cells (Fig 8G). These changes were attenuated with TGF-β1 and AAT co-treatment (Fig 8D and 8H).

### AAT Treatment Attenuated TGF-β1 Mediated Upregulation of Smad3 Phosphorylation in MDCK Cells

**Fig 9:**
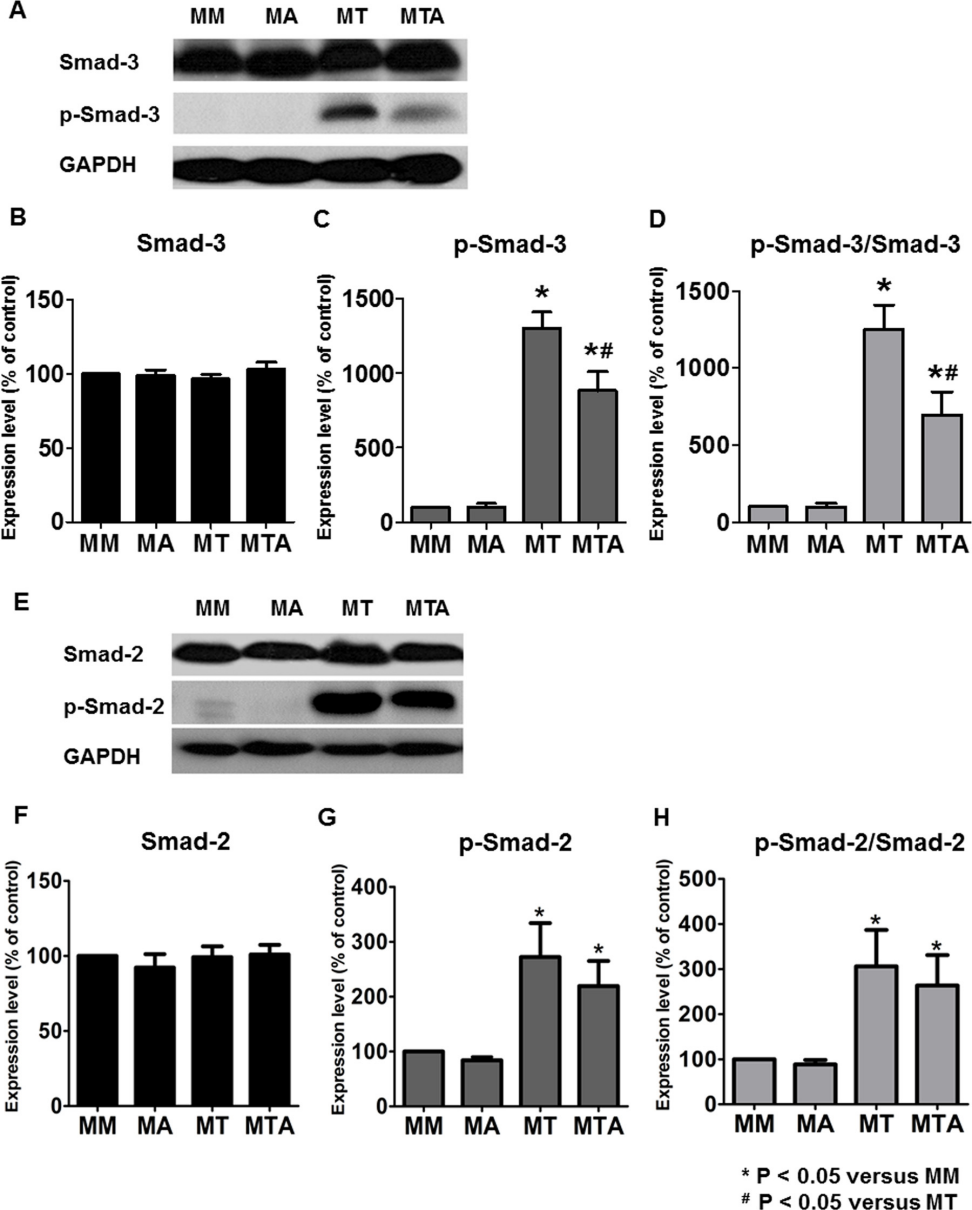
Expression of Smad3 and Smad2 in MDCK cells. Immunoblotting showed that the phosphorylated Smad3 (p-Smad3) level and the ratio of p-Smad3 to Smad3 were significantly decreased in the MDCK cells treated with TGF-β1 and AAT compared to that in MDCK cells treated with TGF-β1 alone (A to D). The level of phosphorylated Smad2 (p-Smad2) and the ratio of p-Smad2 to Smad2 were significantly increased in MDCK cells after treatment with TGF-β1 and showed tendency to decrease in MDCK cells treated with TGF-β1 and AAT (E to H).

We examined whether AAT treatment modulated TGF-β1 induced EMT by inhibiting Smad signaling pathway. The levels of p-Smad3 and Smad3 in MDCK cells were analyzed by immunoblotting. The level of p-Smad3 and the ratio of p-Smad3 to Smad3 were significantly decreased in MDCK cells treated with TGF-β1 and AAT compared to that in MDCK cells treated with TGF-β1 alone. The level of p-Smad2 and the ratio of p-Smad2 to Smad2 were significantly increased in MDCK cells after treatment with TGF-β1 and showed tendency to decrease in MDCK cells treated with TGF-β1 and AAT (Fig 9).

## Discussion

The present study demonstrated that AAT attenuated *in vivo*, renal fibrosis in UUO mouse model. The extent of renal fibrosis was significantly lower in AAT treated UUO group compared to that in UUO or vehicle treated UUO groups. AAT also inhibited TGF-β1 induced EMT in MDCK cells. TGF-β1 exposure increased the expression of α-SMA, vimentin, fibronectin, and collagen I whereas TGF-β1 decreased the expression of E-cadherin in MDCK cells, which was reversed by co-treatment with TGF-β1 and AAT. The attenuation of TGF-β1 induced EMT was mediated by downregulation of phosphorylated Smad3. Our results suggest that the reno-protective effects of AAT could be mediated by inhibition of TGF-β1 induced EMT.

AAT is a potent serine proteinase inhibitor, and its deficiency is associated with the genetic disorder of chronic obstructive pulmonary disease. Recent studies revealed that AAT not only inhibited the serine proteinases secreted by neutrophils, but also controlled the inflammatory process [18,19]. Since the four methionine residues of AAT molecule have powerful antioxidant capacity [20], AAT could suppress a pro-inflammatory response and superoxide production [16]. Some reports showed that AAT reduced chemotaxis of neutrophils by inhibiting interleukin 8 and leukotriene B4 [21,22]. The anti-inflammatory effect of AAT could also be linked to the inhibition of tumor necrosis factor-α (TNF-α), as evidenced in hepatocytes and mice [15,23].

However, whether AAT could modulate the tissue fibrosis, especially in the kidney, is not known. In the present study, UUO mice were used as a renal fibrosis model to evaluate the anti-fibrotic effect of AAT. UUO is a well-established model of progressive loss of renal function, concurrent with the development of tubular atrophy, interstitial inflammation, renal cell apoptosis, and renal fibrosis [24,25]. TGF-β1 is known as a major contributor in the obstruction-mediated renal injury model. Increased level of TGF-β1 is associated with loss of epithelial phenotype and acquisition of mesenchymal phenotype in myofibroblasts, and increased collagen deposition [26]. Since AAT inhibited TNF-α as mentioned earlier, and TGF-β1 was activated by increased TNF-α in UUO model [27,28], we investigated the effect of AAT on TGF-β pathway in renal fibrosis. The *in vivo* protective effects of AAT can be seen from the significant decrease in the renal fibrosis area and attenuation of the changes in EMT markers such as E-cadherin and collagen I. Furthermore, AAT co-treatment with TGF-β1 could reverse TGF-β1 mediated EMT in MDCK cells, suggesting that AAT modulated TGF-β1 pathway to reduce EMT and renal fibrosis.

However, it is difficult to propose the modulation of TGF-β1 pathway as the only pathophysiologic mechanism of reducing the renal fibrosis. Fibrosis is a common pathologic feature of the chronic inflammatory state and thus related to various inflammatory cytokines including IL-1, IL-6, and TNF-α [29]. Considering the known anti-inflammatory activity of AAT, these effects might be associated with the anti-fibrotic effect of AAT. Although we did not evaluate inflammatory cytokines in the present study, it is essential to investigate the cross talk between the anti-inflammatory and anti-fibrotic effect of AAT.

The exact mechanism of how AAT inhibits TGF-β1 induced fibrosis is not known. Among the several TGF-β pathways, Smad3 signaling is considered as a major intracellular pathway, regulating the transcription of target genes involved in renal fibrosis [2]. TGF-β1 stimulation converts Smad3 into phosphorylated form of Smad3 (p-Smad3), which directly binds to the promoter region of collagen genes and inhibits the extracellular matrix degradation [2,29,30]. Our study showed that TGF-β/Smad3 pathway was also inhibited in MDCK cells by AAT treatment. Therefore, in addition to using it for inhibiting serine proteinases, our findings suggest that AAT treatment could be used to inhibit renal fibrosis, at least partially, through modulating TGF-β/Smad3 pathway.

Our study has a limitation as far as the investigation of AAT deficiency in UUO model or TGF-β1 stimulated MDCK cells. Clinically, asymptomatic decrease in AAT levels was detected in healthy individuals [31]. In addition, AAT level is not a representative of the functional activity of AAT, because inactivated forms of AAT were also detected by immunoblotting or enzyme-linked assay [32]. Another limitation of the study is that EMT develops *in vivo* mainly in the proximal tubule, whereas MDCK cells are originated from distal tubule. Therefore, further study is warranted to investigate the effect of AAT on proximal tubule cells. In addition, recent studies reported that the interstitial myofibroblasts which play major role in extracellular matrix remodeling and renal fibrosis were originated from bone marrow, pericytes, or endothelial cells as well as tubular epithelial cells [33,34,35]. To reveal the exact mechanism of AAT to prevent renal fibrosis, more studies with various possible sources of myofibroblasts should be performed to validate our results. Nevertheless, this is the first study to investigate the anti-fibrotic effect of AAT on MDCK cells and the kidneys of UUO mice, making it an additional candidate for renal fibrosis therapy.

In conclusion, AAT, in addition to its anti-inflammatory effect, had an attenuating effect on the progression of renal fibrosis in unilaterally obstructed kidney. AAT also inhibited the TGF-β1 induced EMT in MDCK cells. Our results indicate that AAT could be a potential therapeutic agent to inhibit the pathway of renal fibrosis, at least partly, through the suppression of TGF-β/Smad3 signaling.
